# An Unusual Case of Syphilis With Abdominal Pain

**DOI:** 10.7759/cureus.16806

**Published:** 2021-08-01

**Authors:** Mohsen Dourra, Shiab Mussad, Ana M Capatina-Rata

**Affiliations:** 1 College of Human Medicine, Michigan State University, East Lansing, USA; 2 Internal Medicine, Ascension Providence Hospital - Southfield Campus, Southfield, USA

**Keywords:** syphilis, gastroenterology, infectious diseases, hepatitis, sexually transmitted infections

## Abstract

Syphilis is a multisystem infection caused by *Treponema pallidum*, a common sexually transmitted bacterium. The different stages and associated signs of syphilis are well characterized. We present an atypical case of syphilis in a 27-year-old female with hepatitis and gastritis. The diagnostic criteria for syphilitic hepatitis and gastritis are discussed here, along with endoscopic and clinical findings, treatment, and a brief literature review.

## Introduction

Syphilis cases have been resurging worldwide. In the United States, there were over 115,000 new cases from 2017 to 2018, according to the Centers for Disease Control and Prevention, especially in high-risk populations [1]. Uncommon presentations with hepatitis or gastritis may go unrecognized because it mimics many other pathological processes. This may lead to a delay in diagnosis and treatment. Patients with appropriate risk factors for syphilis should be considered for testing.

## Case presentation

A 27-year-old female presented to the hospital with a three-day history of fever, chills, vomiting, and right upper quadrant pain. She also complained of dark urine and diffuse myalgias and arthralgias. The patient had a medical history of well-controlled asthma. She was sexually active with only one male partner over the past year and was not using barrier protection. The patient denied usage of alcohol, herbal supplements, and medications including acetaminophen, nonsteroidal anti-inflammatory drugs, and proton pump inhibitors. Upon initial examination, she was hemodynamically stable, but tachycardic with a pulse of 123 beats per minute and afebrile. The patient notably had scleral icterus but no jaundice of the skin or rashes. Oral thrush and erythema were found on oropharyngeal examination. The patient had epigastric and right upper quadrant abdominal tenderness. There was no hepatosplenomegaly or ascites. She had erythematous, swollen, and tender proximal and distal interphalangeal joints of the hands bilaterally. No genital skin lesions were identified.

Initial laboratory tests were significant for a white blood cell (WBC) count of 20.55 K/µL, alanine aminotransferase (ALT) of 115 U/L, aspartate transaminase (AST) of 68 U/L, alkaline phosphatase (ALP) of 279 U/L, and total bilirubin of 4.1 mg/dL. Abdominal ultrasound and CT showed cholelithiasis without gallbladder wall thickening, pericholecystic fluid, or other signs of acute cholecystitis. The common bile duct was 3.9 mm in size. A hepatobiliary iminodiacetic acid scan displayed patent cystic and common bile ducts. Duplex ultrasound of mesenteric vessels was unremarkable.The patient tested negative for hepatitis A, B, and C. She was negative for anti-nuclear, anti-smooth muscle, and anti-mitochondrial antibodies. Testing for human immunodeficiency virus, Epstein-Barr virus, and cytomegalovirus among other common viral infections was negative. However, syphilis testing was positive with a rapid plasma reagin (RPR) titer of 1:32 (normal: nonreactive). A liver biopsy was not performed.

Following her positive syphilis test, the patient was treated with three doses of intramuscular penicillin G 2.4 million units. After the first dose of penicillin, total bilirubin, ALP, and other liver enzymes began to improve. Three days following her first dose of penicillin there was marked improvement in her labs as follows: ALP 169 U/L, total bilirubin 1.1 mg/dL, AST 14 U/L, ALT 42 U/L, and WBC 13.40 K/µL.

**Figure 1:**
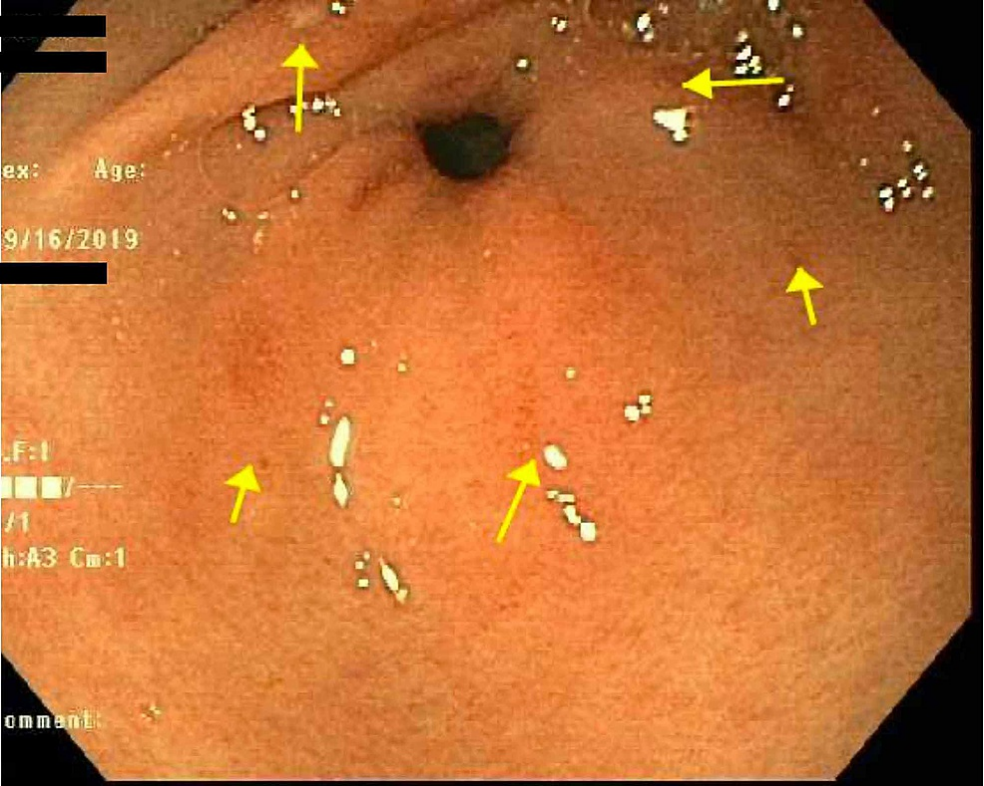
Several superficial ulcers without stigmata of bleeding in the gastric antrum.

The patient was discharged after receiving her first dose of penicillin and after reporting improvement in abdominal pain. However, she returned two days later with similar symptoms, notably epigastric pain, nausea, and vomiting. Extensive workup for gastritis was performed at that time. Esophagogastroduodenoscopy (EGD) was performed and demonstrated multiple nonbleeding duodenal and gastric ulcers with moderate inflammation and erythema. The largest gastric ulcer was 4 mm (Figure 1) while the largest duodenal ulcer was 7 mm. Biopsy showed evidence of erosive gastritis in the gastric antrum. Immunostaining for *Helicobacter pylori* was negative. Staining for spirochetes was not performed at that time. In addition to symptomatic management, the patient received her second and third doses of penicillin. Her transaminases, total bilirubin, and ALP continued trending downward, eventually reaching normal limits, along with a resolution of her symptoms.

## Discussion

Syphilis cases have been increasing at a worrisome rate. In the United States, from 2017 to 2018, there were over 115,000 syphilis cases, according to the Centers for Disease Control and Prevention [1]. Over the same period, the number of primary and secondary syphilis cases, 35,000, increased to the highest number reported since 1991 [1].

Syphilis has various well-recognized presentations. However, hepatitis and gastritis are lesser-known manifestations. Hepatic disease has only been estimated to occur in 0.2% of patients with secondary syphilis, and gastritis has been associated with only 1% of syphilis infections [2,3]. Clinical symptoms are usually nonspecific, making diagnosis challenging. In patients with hepatic syphilis, the most common findings include rash, fatigue, weight loss, scleral icterus, and hepatomegaly [4]. Liver enzymes are abnormally elevated and demonstrate a cholestatic pattern with increased levels of ALP and gamma-glutamyltransferase (GGT) in comparison to AST and ALT [4]. If a liver biopsy is performed, histological findings include periportal hepatocyte necrosis and pericholangiolar inflammation, which may play a role in the elevations of ALP and GGT [5]. Identification of *Treponema pallidum* spirochetes in liver biopsy is considered to be diagnostic [3]. However, liver biopsy is not required for diagnosis as spirochetes are infrequently found. Mullick et al. proposed that a clinical diagnosis of syphilitic hepatitis can be made by satisfying four diagnostic criteria, namely, the presence of abnormal liver enzyme levels, positive serologic testing for syphilis, liver enzymes returning to baseline after treatment, and exclusion of other causes [5].

Our 27-year-old female patient notably presented with abdominal pain, fatigue, and scleral icterus. Serological testing for syphilis was positive with an RPR titer of 1:32. An extensive workup for additional infectious, autoimmune, or other causes was negative. The patient demonstrated a cholestatic liver pattern with an ALP of 279 U/L in contrast to an AST of 68 U/L and an ALT of 115 U/L. Importantly, the patient had a clear improvement in liver enzymes after standard syphilis treatment with penicillin. It is possible that our patient’s pretreatment liver enzymes did not reach higher levels, as reported in other case studies [6], due to prompt diagnosis and treatment. Treatment with penicillin G 2.4 million units has been used effectively in treating hepatitis and gastritis and leading to a rapid resolution of symptoms [4,6,7].

In addition to liver involvement, syphilis has also been implicated as a cause of gastritis. Syphilitic gastritis is hypothesized to be a result of obliterative endarteritis of vessels feeding gastric tissue [7]. Epigastric pain is the most common presenting symptom, found in 92% of patients [7]. Nausea, vomiting, and weight loss are also frequently present. A systematic review by Mylona et al. showed that EGD may reveal multiple findings, including ulcerations, erosions, narrowing, or nodular mucosa. The distal gastric body and antrum are most commonly involved with positive findings in 82% of cases they reviewed [7]. These EGD findings are not entirely specific to syphilis and may also be found with lymphoma or other causes. Histology of gastric lesions often shows evidence of chronic gastritis and infiltration with plasma cells and lymphocytes [7,8]. Detection of syphilis on serology and the presence of characteristic endoscopic and clinical findings are suggestive of the diagnosis. However, the discovery of *T. pallidum* in gastric lesions is considered diagnostic. Silver stain and direct immunofluorescence microscopy are commonly employed techniques on biopsied specimens, along with real-time polymerase chain reaction [3,7]. Our specimens were not tested for spirochetes, so the diagnosis of gastritis could not be definitively confirmed. However, the diagnosis is strongly suspected based on serology, EGD, and clinical characteristics.

## Conclusions

This case highlights the multiorgan involvement of syphilis and how challenging it is to identify syphilis as the culprit during the evaluation of suspected gastritis and hepatitis. Typical syphilis manifestations have been well characterized in the literature. However, uncommon presentations with hepatitis or gastritis are rare and less recognized findings. This may lead to delays in diagnosis and treatment. In patients with risk factors, syphilis infection should be considered in the differential diagnosis of gastritis or hepatitis as delayed diagnosis and treatment can lead to complications such as acute liver failure or gastric ulceration.
